# A SMARCA2 Mutation in the First Case Report of Nicolaides-Baraitser Syndrome in Latin America: Genotype-Phenotype Correlation

**DOI:** 10.1155/2017/8639617

**Published:** 2017-08-29

**Authors:** Ana Isabel Sánchez, Jorge Armando Rojas

**Affiliations:** Instituto de Genética Humana, Facultad de Medicina, Pontificia Universidad Javeriana, Bogotá, Colombia

## Abstract

Nicolaides-Baraitser syndrome (NCBRS) is a rare and well-recognized entity that was first described in 1993, with a prevalence that is currently not known. It is recognized as a distinctive entity, with some variability in its signs and symptoms. The most important characteristics include intellectual disability, peculiar facial features including sparse scalp hair, coarse facial features, low frontal hairline, and microcephaly, and seizures. Additional features may include epicanthic folds, thin upper lip vermilion with thick lower lip vermilion, skeletal abnormalities, and severe language impairment. The disorder is inherited in an autosomal dominant manner caused by de novo mutations in the* SMARCA2 *gene, with most being missense mutations. We report a young adult patient with NCBRS and, to our knowledge, the first case report of the syndrome in Latin America with a confirmed molecular diagnosis and a mild-to-moderate phenotype.

## 1. Introduction

Nicolaides-Baraitser syndrome (NCBRS) is a rare and well-recognized entity that was first described in 1993 by pediatric neurologist Paola Nicolaides and medical geneticist Michael Baraitser in a patient with distinctive features characterized by intellectual disability, peculiar facial features, multiple congenital anomalies, and seizures [1]. After that, further cases were reported [2–5]. In 2009, a follow-up of those patients and the characterization of eighteen more allowed the scientific community to delineate the entire phenotype of NCBRS and made it possible to recognize it as a distinctive entity with some variability in the signs and symptoms [6].

Clinical diagnosis of NCBRS is often challenging because of its progressive nature. There is evidence of a continuum phenotypic spectrum as well a division between a classic or severe phenotype and an atypical or mild form [6]. Additional features may include sparse scalp hair, skeletal abnormalities such as prominence of the interphalangeal joints and distal phalanges because of diminished subcutaneous fat, coarse facial features, microcephaly, and severe language impairment. Autism spectrum disorders have also been diagnosed in these patients [7]. The prevalence is not known; however, to date, around one hundred patients have been described in the literature worldwide [8].

The disorder is inherited in an autosomal dominant manner caused by the novo mutations in* SMARCA2 *gene; most of them are missense mutations and some others have been reported as in frame deletions [9, 10].

Here, we report a young adult patient with NCBRS. To our knowledge, this is the first case report of this syndrome in Latin America with a confirmed molecular diagnosis and a mild-moderate phenotype of an already reported causative mutation. Informed consent was properly obtained from the patients' parents (legal guardians).

## 2. Case Report

This case study describes a Colombian female patient who was referred for medical genetics evaluation at the age of 20 years because of moderate cognitive impairment and dysmorphic features. She was born by vaginal delivery after a full-term 38-week and uncomplicated gestation to a Gravida 2, spontaneous miscarriage 1, 33-year-old mother and her nonconsanguineous 28-year-old male partner. The patient had a birth weight of 3200 grams and a birth length of 49 centimeters. Perinatal distress was described because of prolonged labor; however, perinatal asphyxia was not documented by the doctors.

Motor, speech, and language milestones were delayed in comparison to neurotypically developing peers, with language difficulties being more severe than gross motor and fine motor problems: head up to 45° in prone was achieved at 8 months, body balance control at 11 months, and independent walking at 13 months. However, few two-syllable words were accomplished by the patient at the age of 5 years and roughly two-word phrases at 11 years old. Since childhood, the patient had undergone rehabilitation language therapies; however, at the time of examination, intelligible speech with important dyslalia was noticed. Despite her impairment for linguistic skills, patient was able to socialize adequately with her parents and even with strangers. She was also able to follow very simple instructions, and social smile was evident in her. She needs assistance for activities of daily living.

At 18 months of age, she developed generalized epilepsy that was well controlled by antiepileptic medication (Carbamazepine), with the last crisis documented at the age of 5 years. Brain abnormalities were not recognized on MRI. Irritability and aggression had been present since childhood; however, these behavioral disturbances gradually decreased in severity and frequency with psychiatric management and medication (Risperidone). Severe thoracic scoliosis required surgical intervention at the age of 9 years, with an outstanding outcome, but general health was good otherwise.

**Figure 1:**
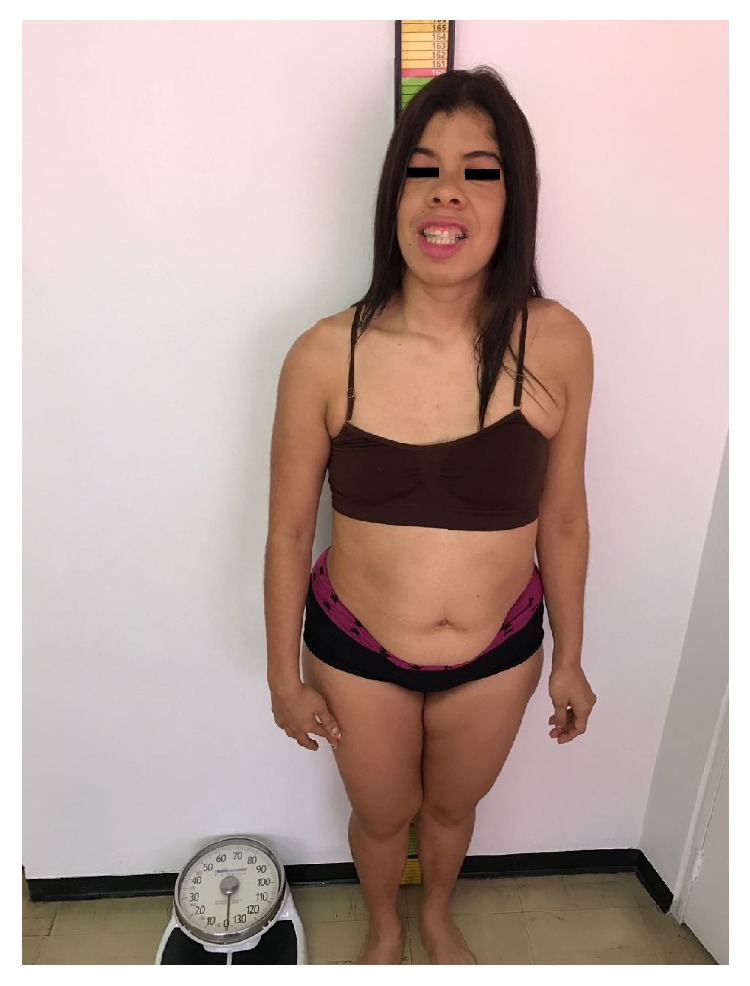
Clinical photograph of the patient showing features of overweight and normocephalic head.

Physical examination revealed a slightly overweight patient with a normocephalic head, as seen in Figure 1. Height was 156.5 cms (*p* 60), weight was 69 kg (*p* 90–95), and cephalic perimeter was 54 cms (*p* 50–75).

**Figure 2:**
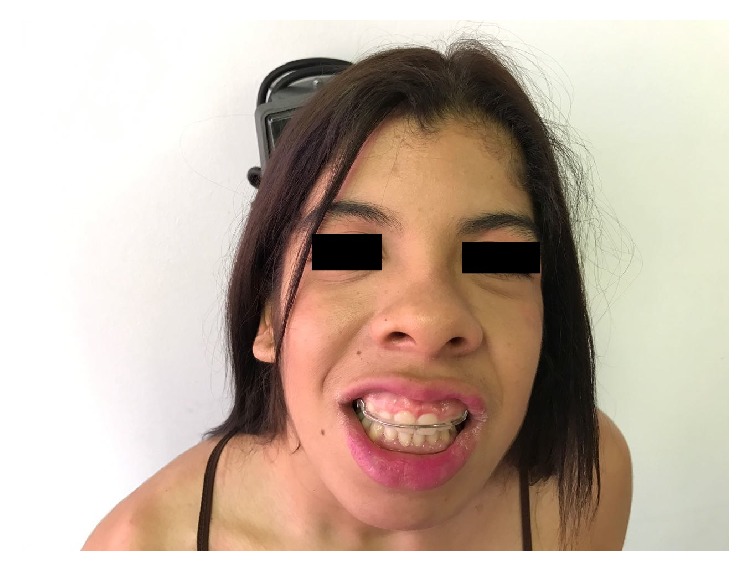
Clinical photographs of the patient demonstrating a coarse face, some sparse hair, low frontal hairline, broad eyebrows, long eyelashes, broad nasal bridge with thick alae nasi, upturned nasal tip, long and broad philtrum with a wide mouth, gum hypertrophy, and a thick lower vermilion.

**Figure 3:**
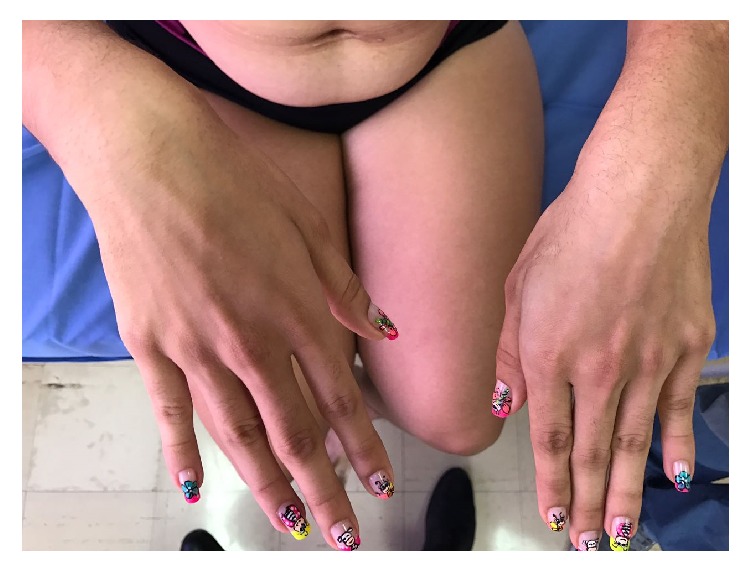
Clinical photographs of hands demonstrating prominent interphalangeal joints.

She had mild coarse face, some sparse hair, low frontal hairline, down slant palpebral fissures with sagging periorbital skin, epicanthic folds, broad eyebrows, and long eyelashes. A broad nasal bridge, upturned nasal tip and long philtrum with a wide mouth, gum hypertrophy, and thick lower vermilion were also seen in this patient (Figure 2). A side view of the patient's face can be seen in Supplementary Material S1 available online at https://doi.org/10.1155/2017/8639617. The distal phalanges of the fingers and toes were narrow, with prominent interphalangeal joints (Figure 3). Finger nails and toe nails were slightly hyperconvex. She also had eczema, particularly involving the trunk, arms, and legs.

Because of progressive coarsening of facial features and moderate intellectual disability due to a brief intelligence quotient of 55, consistent with the diagnosis of moderate intellectual disability, biochemistry enzyme assays were performed and ruled out the diagnoses of Sanfilippo syndrome, Hurler syndrome, and Maroteaux-Lamy syndrome. Urine amino acids, organic acids, and analysis of urinary oligosaccharides and glycosaminoglycans were normal by chromatography. Conventional cytogenetic studies and CGH microarray also gave normal results. Heart ultrasound and hearing tests showed no abnormalities.

Whole exome sequencing analysis on DNA extracted from the patient's blood was performed using NGS technology (Illumina®), with later confirmation by Sanger sequencing. A heterozygous missense mutation in the* SMARCA2* gene was reported, specifically c.2564G>A (p.Arg855Gln). The patient's clinical phenotype of particular facial features, diminished language, cognitive impairment, and seizures, along with the* SMARCA2* mutation, was consistent with NCBRS.

## 3. Discussion

We report a patient that was diagnosed with NCBRS at the age of 20 years by documentation of a* SMARCA2* pathogenic mutation and by clinical examination. Patient's first consultation to genetics clinics was when she was an adult, so, as geneticists, we were not able to see the progression in the phenotype, as NCBRS patients usually have. Additionally, because patient's peculiar faces were really subtle, and because cognitive impairment is a major symptom of many syndromes, including those of the spectrum, like Coffin-Siris syndrome, it was difficult at age of 20 years to make the diagnosis only by clinical suspicion. This is why, and after excluding anomalies in conventional cytognetics studies and in CGH microarray, we decided to perform whole exome sequencing, as the patient met criteria to do this genetic test. Besides this, it has to be said that her late diagnosis was due to the fact that the phenotype in the disorder is slightly progressive and some characteristics appeared only at older ages. At younger ages, facial features are subtle and although there is global developmental delay, this is also a relatively common neurological condition that can be caused by different genetic and nongenetic conditions; therefore, it is not specific for NCBRS.

Apart from moderate-to-profound intellectual disability [11], neurological involvement in the syndrome may include seizures in up to two-thirds of patients, like in this one [12]. However, this female individual did not present with all of the features of the typical NCBRS craniofacial phenotype. She did not show the triangular shape of the face, the microcephaly, and the thin vermilion of the upper lip, nor the short stature that has been distinctively described in the disorder.

The identification of clustered mutations in a large number of patients with NCBRS supports the idea that this syndrome is a distinct clinical entity [13]. The mutation found in this patient (p.Arg855Gln) was previously reported and has been predicted to be deleterious by various in silico pathogenicity prediction tools (SIFT, PolyPhen, and MutationTaster). It is located in exon 18, which is part of the major SNF2 ATPase domain of the* SMARCA2* gene, specifically in the II helicase‐related sequence motif of the Helicase ATP-binding domain [9, 13].

The SMARCA2 gene is located on 9p24.3 and spans 34 exons, encoding a 1590 amino acid protein. This gene is a member of the Swi2/Snf2 family, which share an helicase-like ATPase domain that includes a region with seven canonical helicase‐related sequence motifs involved in ATP‐binding/hydrolysis as well as in DNA‐binding and play different roles in cellular differentiation and transcription control by remodeling nuclear chromatin [9, 14, 15]. This ATPase domain is highly functionally conserved between species [9, 13]. Considering that whole deletions of the gene do not cause the NCBRS phenotype and that mutations in this ATPase region are missense but not truncating mutations, it has been postulated that mutations identified in NCBRS do not lead to haploinsufficiency but rather have a dominant negative or gain‐of‐function effect [9, 13].

The vast majority of mutations causing NCBRS are located in the ATPase domains of exons 15–25 of this gene [9, 16, 17]; however, atypical variants outside the ATP domain have also been described in individuals with typical NCBRS, highlighting the broad spectrum of the syndrome and helping to elucidate more genotypes and phenotypes [16].

There is a correlation between the severity of cognitive impairment and epilepsy, language impairment, short stature, and microcephaly; it is known that patients with severe or moderate intellectual disability are most likely to have short stature, microcephaly, and seizures and that patients with seizures also have a higher prevalence of microcephaly [9]; however, this is not completely accurate in our patient. Individuals with mutations affecting the C-terminal helicase region from the SMARCA2 ATPase domain tend to have severe intellectual disability and a higher chance of developing seizures [9]. However, although our patient had significant cognitive impairment and epilepsy, the mutation reported was located in the Helicase ATP-binding domain.

This case report suggests that the mutation found may be strongly associated with speech problems, intellectual disability, and epilepsy, more than with specific craniofacial features and physical phenotype.

## Supplementary Material

Side view of the patient's face in S1.
