# Supplementary figures and images for: A SMARCA2 Mutation in the First Case Report of Nicolaides-Baraitser Syndrome in Latin America: Genotype-Phenotype Correlation
Source: Case Rep Genet. 2017 Aug 29;2017:8639617. doi: 10.1155/2017/8639617 (PMC5602489; doi:10.1155/2017/8639617)

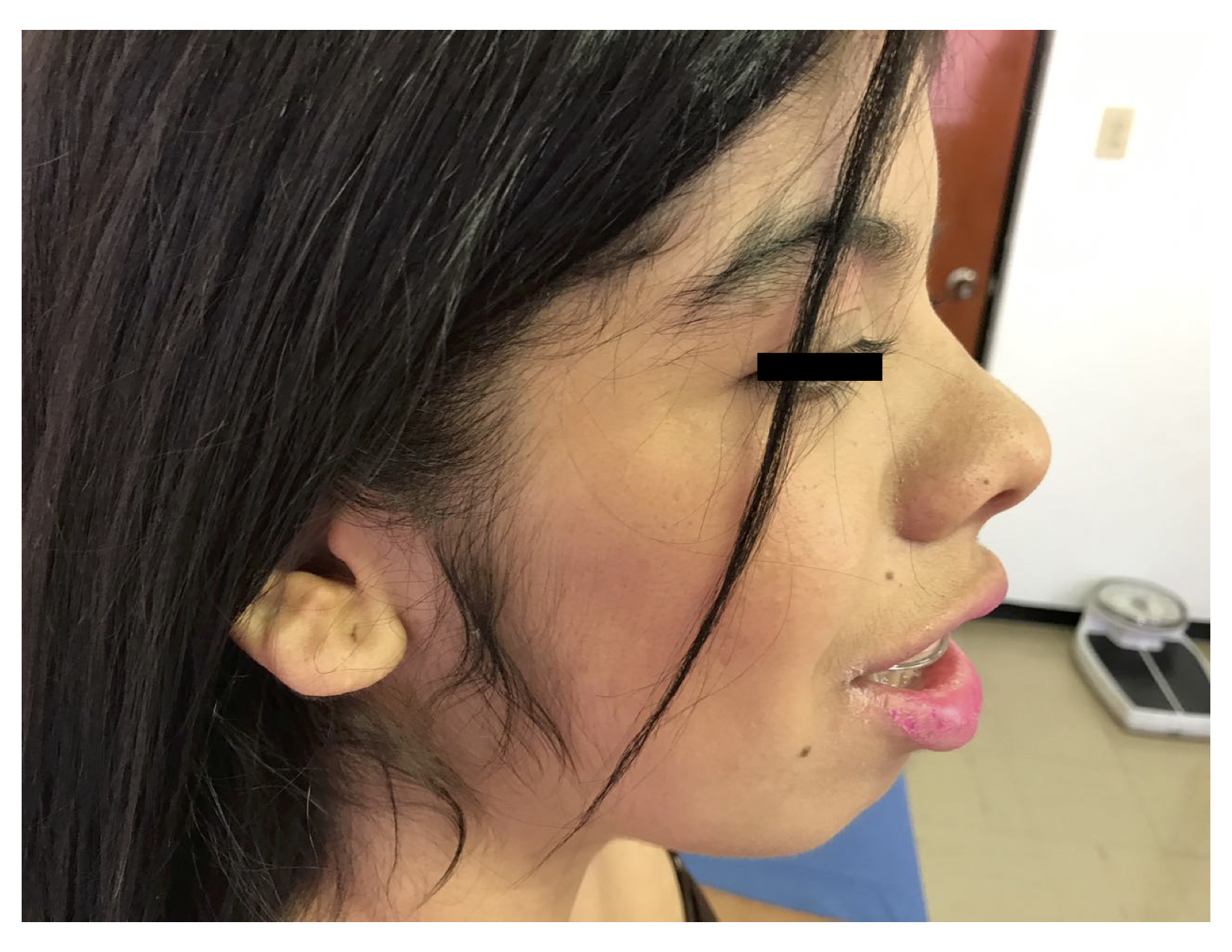

Supplement: Supplementary file 1 — Side view of the patient's face in S1. [file 8639617.f1.tiff]
